# Mechanical Problem Solving in Goffin’s Cockatoos—Towards Modeling Complex Behavior

**DOI:** 10.1177/10597123241270764

**Published:** 2024-08-15

**Authors:** Manuel Baum, Theresa Rössler, Antonio J. Osuna-Mascaró, Alice Auersperg, Oliver Brock

**Affiliations:** 1 Robotics and Biology Laboratory, Technische Universität Berlin, Berlin, Germany; 2 Science of Intelligence (SCIoI), Cluster of Excellence, Berlin, Germany; 3 Comparative Cognition, Messerli Research Institute, Medical University of Vienna, 587245 University of Veterinary Medicine Vienna, University of Vienna, Vienna, Austria; 4 Department of Cognitive Biology, 27258 University of Vienna, Vienna, Austria

**Keywords:** Problem solving, parrots, decompositional analysis, lockbox

## Abstract

Goffin’s cockatoos (*Cacatua goffiniana*) can solve a diverse set of mechanical problems, such as tool use, tool manufacture, and mechanical puzzles. However, the proximate mechanisms underlying this adaptive behavior are largely unknown. Similarly, engineering artificial agents that can as flexibly solve such mechanical puzzles is still a substantial challenge in areas such as robotics. This article is an interdisciplinary approach to study mechanical problem solving which we hope is relevant to both fields. The behavior we are studying results from the interaction between a complex environment (the lockbox) and different processes that govern the animals’ behavior. We therefore jointly analyze the parrots’ (1) engagement, (2) sensorimotor skill learning, and (3) action selection. We find that none of these aspects could solely explain the animals’ behavioral adaptation and that a plausible model of proximate mechanisms must jointly address these aspects. We accompany this analysis with a discussion of methods to identify such mechanisms. At the same time, we argue, it is implausible to identify a detailed model from the limited behavioral data of just a few studies. Instead, we advocate for an incremental approach to model building in which one first establishes constraints on proximate mechanisms before specific, detailed models are formulated. To illustrate this idea, we apply it to the data presented here. We argue that as the field attempts to find mechanistic explanations for increasingly complex behaviors, such alternative modeling approaches will be necessary.

## 1. Introduction

Flexibility is one key element that allows animals to solve novel tasks or new variants of previously encountered tasks (Reader & Laland, 2003). Such flexible adjustment is currently a key challenge to researchers who engineer machine behavior, such as in robotics. Thus, uncovering the processes that are involved in learning and adapting to new tasks will not only benefit behavioral biology, but also benefit approaches to engineer machine behavior.

**Figure 1.**
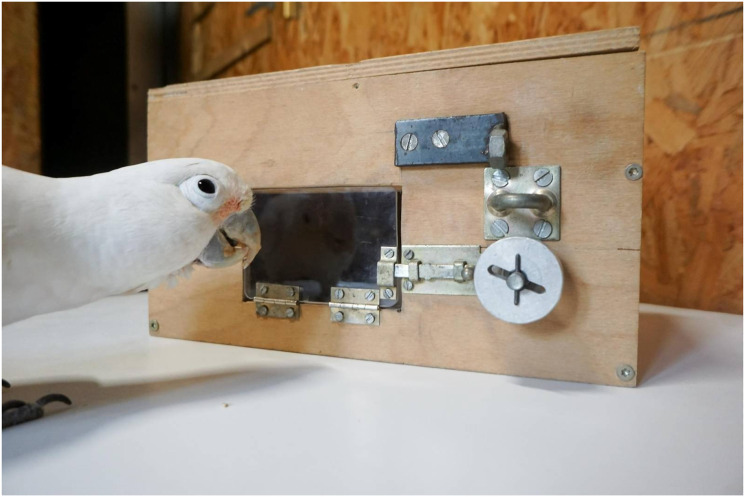
Zozo in front of the lockbox, a baited mechanical puzzle which reveals the reward only after a sequence of mechanisms is unlocked. The configuration is as presented to the subjects; window closed, bar in locked position and wheel attached with the opening horizontal so as to be perpendicular to the pin (already slightly rotated by Zozo in this picture).

A species that is impressively flexible in its behavior is the Goffin’s cockatoo (*Cacatua goffiniana*; Ibáñez de Aldecoa et al., 2022; Laumer et al., 2016). These parrots show extensive object manipulation, recombination, and haptic exploration (e.g., Auersperg et al., 2014; Auersperg et al., 2015). Furthermore, they skillfully solve a wide range of mechanical problems (e.g., Habl & Auersperg, 2017; Rössler et al., 2020), such as the lockbox (Auersperg et al., 2013). Lockboxes are mechanical puzzle boxes that require the opening of a sequence of locks in the correct order to access a reward (see Figure 1). Lockboxes have also served as experimental scenarios in robotics (Baum et al., 2017), providing an interesting and bi-directional route of transfer of results between biological and artificial agents.

In this article, we study of mechanical problem solving in Goffin’s cockatoos jointly as behavioral biologists and roboticists. Driven by the roboticists’ desire to build real robots, we evaluated the realizabilty of current modeling practice in behavioral biology. We arrived at the conclusion that there is a significant gap between the models commonly formulated, which predominantly *describe* behavior, and those models that would be sufficient to *produce* that behavior. As a first step to close this gap and to advance modeling practices to *produce* behavior, we employ two means: First, we perform a decompositional analysis of three birds’ learning process in a lockbox task. Such an analysis provides a more grounded insight into how this behavior can be reproduced, based on a dissection into different behaviorally relevant factors. Secondly, we also identify from our behavioral observations constraints on mechanisms that might enable the cockatoos’ behavioral flexibility.

### 1.1. Decompositional analysis

Behavior results from a multitude of heterogeneous processes that can substantially interact (e.g., Acerbi et al., 2022; Bandini et al., 2020; Biondi et al., 2022; Griffin, 2016; Griffin & Guez, 2014, 2016; Schierwagen, 2012). Thus, task-relevant changes in behavior can happen because of changes either in single processes or in their mutual interactions. Therefore, it is crucial that behavioral analysis jointly analyzes the different processes that may be the basis of adaptation. We perform such a joint analysis of different adaptive factors. We analyze how engagement, sensorimotor skills, and task-solving strategy change over time as Goffin’s cockatoos learn to solve a mechanical puzzle task. It is common in robotics and AI to differentiate between skills and strategies, the latter determining the sequence of skills to be executed (Colledanchise & Ögren, 2018; Egerstedt, 2000). From biological studies, it is known that engagement can have a significant impact on task performance (reviewed in Griffin & Guez, 2014).

Therefore, we investigate the aforementioned factors: engagement, sensorimotor skill, and task-solving strategy. While other factorizations would be possible, we discovered that this factorization is an exact decomposition of the animals’ task performance and that performance can be reconstructed as the mathematical product of these factors. Other studies have also applied such joint analyses (for a recent example, see Biondi et al., 2022). A distinct difference of our approach is that we did not test a-priori hypotheses based on knowledge about behavior, but instead aimed to find factors enabling the construction of artificial models.

### 1.2. Identification of constraints

Behavioral biologists build increasingly accurate understanding of behavior by iteratively eliminating explanations that are inconsistent with the data. In this spirit, we will identify the set of models that are consistent with all observations, but additionally we would like to select the one that is also a plausible mechanical realization, capable of producing the modeled behavior (as e.g., conducted in Baum et al., 2022). However, this approach suffers from the many-to-one problem (Taylor et al., 2022), which describes that there are potentially many different mechanistic models that can explain the same behavioral data. Thus, we do not think it will be effective to gather sufficient data, even across the whole community, to perform such inference.

Instead, we suggest collecting *constraints* on proximate mechanisms. Constraints are statements that narrow down the set of possible mechanisms, without directly specifying the mechanism. They represent an intermediate stage between raw data and fully specified mechanistic models. While not as sharply defined as concrete model mechanisms, they are more mechanistic than pure observational data. We suggest that such an intermediate level of modeling is essential to bridge the gap between biological data and implementable algorithmic models that can be executed on robots. After we present experimental data below, we will list constraints that mechanisms should likely fulfill to explain the lockbox-solving behavior described below.

## 2. Setting up the bird experiment

### 2.1. Subjects and housing

We tested three adult Goffin’s cockatoos (two males, Zozo and Muki; one female, Fini. They were 8, 7, and 11 years of age, respectively). One male, Zozo, had been initially exposed, but not included, in the previous 5-lock study (Auersperg et al., 2013, data collected in 2011). At the time he was a 1-year old juvenile and was not able to solve the preliminary steps required to enter the experiment, namely, opening the window and sliding the bar (see Pre-Training below). Muki and Fini had not taken part in the 5-lock study. The birds were housed in a group aviary with 13 other Goffins. Their diet consists of a variety of seeds, fruits, vegetables, eggs, and nutritional supplements. The aviary consists of a spacious outdoor compartment (150 *m*^2^ ground area, 3−5*m* height) and an indoor part (45 *m*^2^ ground area, 3−6 *m* height). The latter is heated to 20°*C* during winter. Testing was conducted individually in an adjacent, visually occluded test compartment (9 *m*^2^, 3 *m* height). All birds regularly take part in behavioral studies, in which they have to extract food from different puzzle boxes (e.g., Auersperg et al., 2016; Auersperg et al., 2018; Beinhauer et al., 2019; Habl & Auersperg, 2017; Lambert et al., 2021; Laumer et al., 2017; Osuna-Mascaró et al., 2022; Rössler et al., 2020).

### 2.2. Ethics

The experiment was not invasive and was therefore not classified as animal experiments in accordance with the Austrian Animal Experiments Act (TVG 2012). The task itself used an appetitive protocol based on the parrots’ interest in the rewards and the task. The birds were not food deprived prior to the experiment. All the birds were hand-raised, derived from European breeders, and have full CITES certificates. They are also officially registered according to the Austrian Animal Protection Act (sect; 25—TschG. BGBl. I Nr. 118/2004 Art. 2. 118) at the district’s administrative animal welfare bureau (Bezirkshauptmannschaft St. Pölten Schmiedgasse 4–6, 3100; St. Pölten, Austria).

### 2.3. Apparatus

We used part of the lock apparatus described in (Auersperg et al., 2013), termed the “lockbox.” It consisted of a wooden, rectangular box with a transparent acrylic window (see Figure 1). The window could be blocked with a sequence of mechanical devices. In the present experiment, (1) access to the food reward was impeded by the door that was blocked by a bar that had to be pushed to the right to open the door. Moving the bar could only be done after (2) displacing a wheel forward on its axis, but shifting the wheel was only possible after (3) a partial rotation, so that a slot in the wheel was aligned to a pin in its axis (see Figure 1). Birds were first pre-trained with the opening mechanisms of the door and the bar. The experimental treatment started when the wheel was attached for the first time. The wheel’s slot was perpendicular to the pin at the beginning of each session.

### 2.4. Procedure

#### 2.4.1. Habituation

Like most parrot species, Goffin’s cockatoos are both explorative and neophobic (Greenberg & Mettke-Hofmann, 2001; O’Hara et al., 2017). To reduce neophobic reactions towards the lockbox, we first habituated the birds to the apparatus: We presented it with sunflower seeds (medium quality rewards) on top and around the lockbox and placed a piece of cashew nut (a high quality reward) inside the box while leaving the window open. This continued until they willingly fed on the cashew without any signs of neophobia. Additionally, and to reduce neophobic reactions towards the wheel, subjects were fed sunflower seeds from on top of the wheel detached from the apparatus and outside the experimental room, in the group aviary.

#### 2.4.2. Pre-training

The lockbox was presented to the subjects with a piece of cashew in the door compartment, which was already opened at this stage. Once they readily took the cashew, the door was gradually closed in repeated presentations, until the birds were skilled in opening it. Subsequently, the door was fully closed and blocked by the bar. From that stage onwards, the subject had to push the bar to the right before being able to open the door. During this training, the experimenter was allowed to guide the bird’s attention to the contact point and presented how the mechanisms worked. These pre-training sessions were conducted until each bird reliably shifted the bar and opened the door within 1 minute and without interference by the experimenter. One bird, Fini, showed aversive reactions in the first test session, when the wheel was attached to the lockbox for the first time. After 10 minutes, we aborted the session and conducted an additional pre-training/habituation session with the detached wheel 20 *cm* away from the lockbox and baited with a piece of cashew. On the next test day Fini was re-tested in test session 1.

#### 2.4.3. Test

In test sessions, the configuration was as presented in Figure 1 and described in Sec. Apparatus. Sessions lasted 15 min or until the reward was reached, whichever happened first. We conducted 12 sessions per bird in February–March 2018, during which all three birds became competent in completing the sequence within a session. Two of the birds completed 17 additional sessions in September–November (sessions 13–29; the third subject lost motivation to participate). During test sessions, the experimenter sat next to the birds, wearing mirrored sunglasses and avoiding lateral head movements. Her only interventions were to occasionally tap on the center of the table to draw the subject’s attention to the task or reposition the bird back onto the centre of the table, if it flew off. Just before the additional sessions (13–29), the birds were fed five small cashews from inside the open box and once from the wheel while detached from the apparatus, to re-acquaint them with the apparatus. One subject, Muki, successfully removed the wheel in session 7 but then failed to shift the bar in the remaining 12 min of that session. This subject received two sessions that started without the wheel, namely, continuing from the state it had reached in session 7—until it successfully solved the lockbox.

### 2.5. Behavioral coding

Test sessions were recorded with two video cameras and the behavior was annotated using the open-access software BORIS (Behavioral Observation Research Interactive Software; version 7; Friard & Gamba, 2016). We coded the duration of each session and all defined actions taken towards the lockbox (i.e., physical contacts with each lock, see detailed ethogram in Supplementary^1^).

### 2.6. Data analysis and implementation

We performed our data processing and visualization using custom python scripts. The specific steps of analysis are explained in the next section. Implementations as well as data have been uploaded to the OpenScienceFramework^1^ (Foster & Deardorff, 2017). As a first step, these scripts converted the data in our BORIS annotation project files to pandas (v 1.1.2; Wes McKinney, 2010) data-frames. We then used numpy (v 1.22.3; Harris et al., 2020) for numerical operations on the data and finally plotted the data using matplotlib (v 3.1.2; Hunter, 2007) and seaborn (v 0.11.0; Waskom, 2021).

## 3. Decompositional analysis of adaptation in the lockbox task

When animals learn to solve a challenging task, such as the lockbox, behavioral adaptation may occur in various aspects. In the following, we analyze the animals’ time to solution and three key factors it hinges on: their strategy, sensorimotor skill, and engagement. We demonstrate that these three factors’ combined effect determines the time to solution.

### 3.1. Time to solution

**Figure 2.**
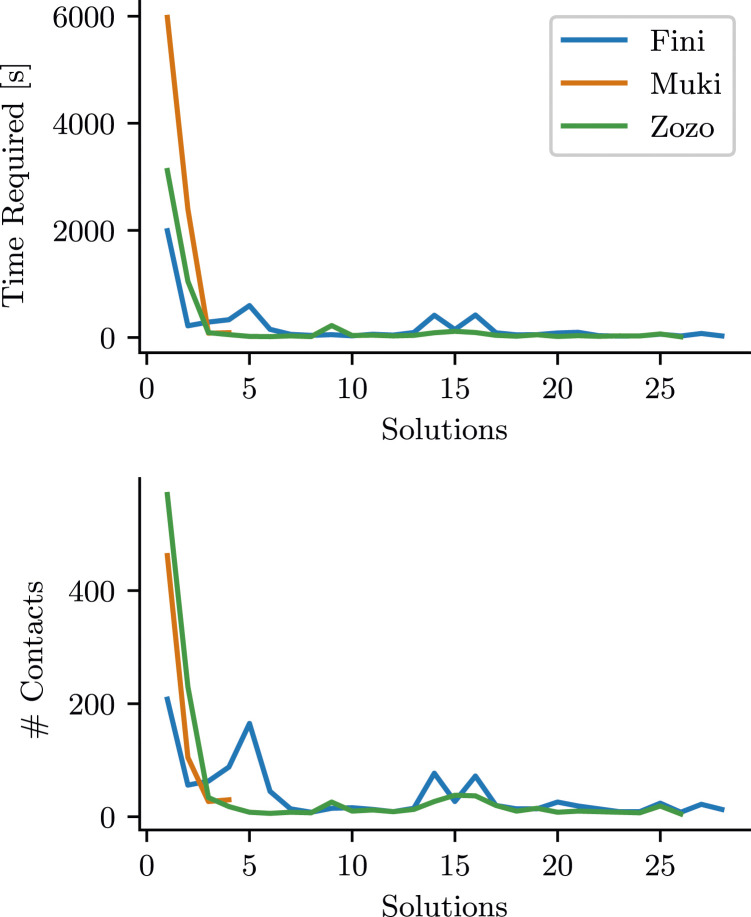
The effort each individual required per solution; plotted as cumulative time duration (top) and the amount of contacts (bottom) needed between consecutive solutions.

Our initial step in the analysis involved quantifying task performance, which we measured as “time-to-solution” (*T*)—essentially, the time it took to successfully complete the lockbox task. In Figure 2, we plotted *T* for each bird across consecutive solutions, alongside the number of actions performed within the corresponding time frame. Fini and Zozo managed to solve the lockbox in less than an hour during their first attempts, while Muki needed about one and a half hours. However, after the first success, the time to solve the problem dropped drastically for all birds and showed little variation. The plots for number of actions show a similar pattern. This rapid reduction in the effort required to solve the task is a distinctive feature of these learning curves.

### 3.2. Mistargeting of actions

As a first factor that may influence *T*, we analyzed the birds’ capacity to choose actions that effectively contributed to solving the lockbox. In principle, birds could utilize a wide range of actions as long as these actions fulfill the necessary steps to open the lockbox (align T-bar with slot in wheel, remove wheel by pulling it over the T-bar, push bar to the side, open door). Creating a precise model of these actions would be challenging, as it would involve distinguishing between pushing, pulling, and other actions on the same object. Thus, we evaluated the quality of action selection by comparing the number of contacts the birds made in functional versus non-functional locations.

Specifically, at the start of the first session and after each consecutive solution, the “correct” behavior was to direct all actions to the wheel until it was removed, then to act on the bar, and finally on the door. A measure of learning could then be the proportion of total actions addressed to the correct part in each state. To compute this, we defined *n*_*w*|*w*_ as the number of actions a bird performed on the wheel in the state when the wheel needed to be removed, and defined *n*_*a*|*w*_ as the total number of all actions the bird performed in that state. Similarly, we defined *n*_*b*|*b*_ and *n*_*a*|*b*_ as the correct and total number of actions when the bar should have been touched, as well as *n*_*d*|*d*_ and *n*_*a*|*d*_ as the correct and total number of actions in the state when the door should have been touched. The measure *M* = *n*_*a*_/*n*_*c*_ = (*n*_*a*|*w*_+*n*_*a*|*b*_+*n*_*a*|*d*_)/(*n*_*w*|*w*_+*n*_*b*|*b*_+*n*_*d*|*d*_) then captured the degree to which the birds make contact in non-functional locations. We termed this measure *M* Mistargeting-of-Actions. Its reciprocal, *p* = 1/*M* would represent the probability to make contact with a functionally relevant part of the lockbox. Regarding allocation, a value of *M* = 1 indicates that the bird exactly followed the right allocation strategy, while *M* ≫ 1 indicates that the bird rarely made contact with those parts of the lockbox it should move.

**Figure 3.**
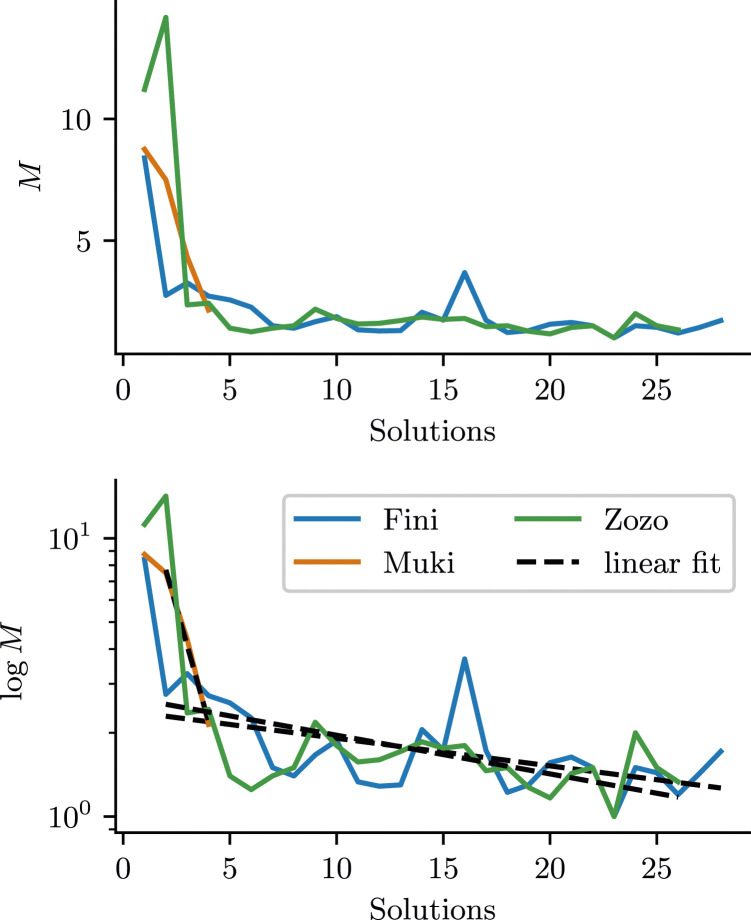
Mistargeting of Actions *M* (top) and log(*M*) (bottom); Large values of *M* indicate many mistargeted actions and *M* = 1 would correspond to perfectly task-directed actions. We fitted a linear function to log  *M* for solutions after the first one, to assess if there is ongoing improvement after the first solution.

Figure 3 shows how *M* and log(*M*) evolve as a function of the number of solutions. *M* shows that the birds significantly improve in the first one or two solutions. This makes *M* an important exploratory factor for the birds’ improvement in time-to-solution (*T*). The plot of log(*M*) can reveal nuanced differences between small values of *M*. For all data *after the first solution*, we fit linear functions to log(*M*) (the functions are linear in log space). The falling slopes of these linear models indicate that also after the first, initial drop in *M*, there is ongoing adaptation.

### 3.3. Manipulation effort

**Figure 4.**
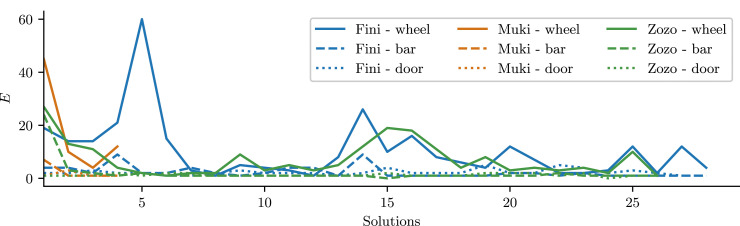
Number of correctly targeted actions required to reach consecutive solutions. This plot shows the number of actions *E* required to open a lock, only counted in states where the lock can actually be opened. This filtering is performed to differentiate sensorimotor capabilites (*E*) from action selection (*M*).

A second factor that might impact time-to-solution is the birds’ sensorimotor expertise at opening the individual locks. We quantified this through the amount of correctly addressed actions *n*_*w*_ required to remove the wheel, *n*_*b*_ required to open the bar, and *n*_*d*_ required to open the door. These are depicted in Figure 4. The figure reveals that as the birds progress through the task in subsequent attempts, they required fewer actions to complete individual locks compared to their earlier sessions. Albeit this trend was non-monotonous. The total number of functionally relevant contacts *n*_*c*_ accumulated as the sum *n*_*w*_ + *n*_*b*_ + *n*_*d*_.

It is worth noting specific observations from individual sessions. The data for Fini, as shown in Figure 4, differ from those of the other two birds in that this subject showed two sessions (5th and 14th) where the number of wheel-directed actions used to remove the wheel was significantly larger than expected for that level of expertise. In these sessions, Fini displaced the wheel forward, but without rotating it sufficiently. As a result, the slit was misaligned to the pin, preventing the wheel from being fully removed. We cannot distinguish whether this was simply an “error” or exploratory behavior, but such events are likely part of the learning process: the birds need to fail in order to learn that both forward displacement and rotation are necessary for success.

### 3.4. Engagement/inter-contact interval

A third factor that influences time-to-solution is the level of engagement of animals with the task posed to them. Often, an animal’s performance in a task is not solely affected by its competence but also by motivational factors (for discussions, see e.g., van Horik & Madden, 2016; Biondi et al., 2022). Occasionally, an individual that has previously demonstrated competence in solving a task may “lose interest” and either remain inactive or engage in behavior unrelated to the task at hand. It is likely that such factors evolve alongside the learning process, and neglecting them could lead to overlooking vital information. In order to avoid such issues, we evaluate the animals’ engagement with the task.

**Figure 5.**
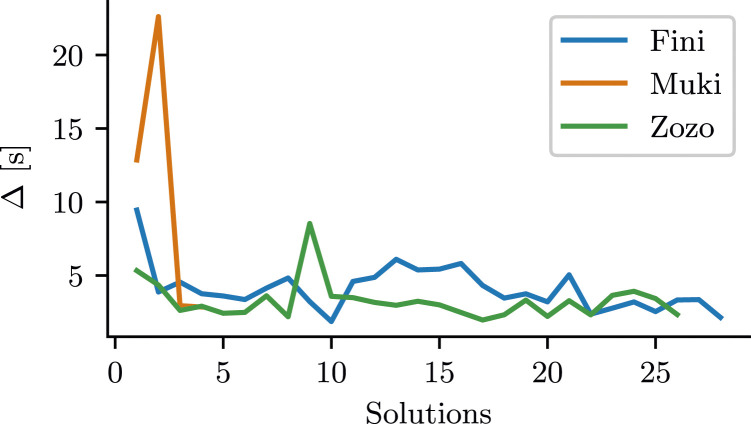
Engagement of the birds with the lockbox. Engagement was operationalized as Inter-Contact Interval (Δ), the average time-duration between physical interactions with the lockbox. Lower Δ corresponds to higher engagement.

To assess the birds’ engagement with the puzzle, we measured the average time duration between timestamps where the birds initiated contact with the lockbox. We refer to this measure as Inter-Contact Interval and denote it Δ, where Δ = *T*/*n*_*a*_. Lower Δ values correspond to a lower time to solution, all other factors being equal. In Figure 5, we plotted engagement as a function of the number of solutions. Relative to their first solution, all 3 birds showed an increase in engagement (signified by a decrease in Δ) in subsequent solutions, except for Muki’s second and Zozo’s ninth solution where Δ is higher than for their first solutions. Table 1 shows data for the first and last solution of each bird. It displays Δ alongside the number of contacts and time-to-solution used to compute the metric.

**Table 1.** Increased engagement measured as decrease in *inter-contact interval* Δ.

		Duration(s)	# Contacts	Δ
Fini	First solution	1993.94	208	9.62
	Last solution	27.92	13	2.10
Muki	First solution	5978.41	462	14.29
	Last solution	88.554	30	2.95
Zozo	First solution	3117.764	570	5.46
	Last solution	11.72	5	2.34

### 3.5. Joint analysis of factors M, E, and Δ

In order to understand how the animals in our study learn to solve the lockbox, we found it important to jointly examine the three aspects: strategy, sensorimotor skill, and engagement. This decomposition *T* = *M*∗*E*∗Δ is a mathematical identity, as becomes apparent in equation (1)

(1) $$T=n_{a}*\frac{T}{n_{a}}=\frac{n_{a}}{n_{c}}*n_{c}*\frac{T}{n_{a}}=M*E*\Delta$$

However, beyond this mathematical reformulation, there are compelling arguments for adopting this decomposition. As discussed in the introduction, intelligent behavior arises from a combination of heterogeneous processes that interact and rely on each other. The three factors we propose as a (non-exlusive) explanation for task performance may serve as examples of such heterogeneous, interdependent processes. An agent capable of solving the lockbox requires a minimum level of competence in action selection strategy and mechanical skill, in addition to being engaged with the puzzle. The multiplicative relation that results in *T* is also logically consistent with the insight that the bird can divide *T* by half either by learning to remove the wheel with half as many actions (adaptation in *E*), by mistargeting half as many actions (adaptation in *M*), or by working on the puzzle twice as fast (adaptation in Δ). These are extreme examples, combinations thereof are also possible.

**Figure 6.**
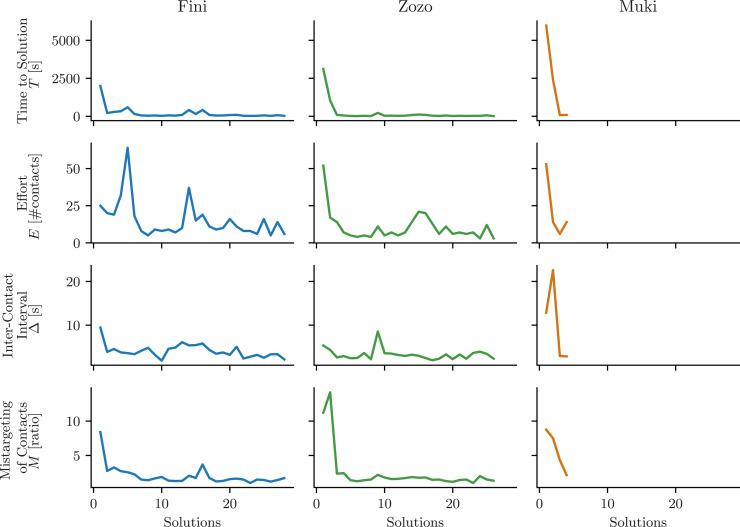
Task performance and three different factors that may contribute to it: Effort *E*, Inter-Contact Interval Δ, and Mistargeting of Contacts *M*. Each measure is plotted individually per bird. This plot provides a visual summary of the data presented in Figures 2–5.

For each bird, the three factors and *T* developed differently over time. This becomes apparent in Figure 6, which provides a visual summary of these measurements. In all three birds, *T* decreased to low values after a few solutions, with the most significant drop in *T* occurring between the first and second solution. Fini demonstrated the most noteworthy change in Δ and *M*, while Zozo showed most change in *E* and *M*, while Muki showed relevant decreases in *E*, Δ, and *M*.

These results underscore that an analysis of adaptation in challenging tasks should strive to encompass multiple of the involved adaptive processes. It would not be possible to reconstruct *T* solely from just one of the analyzed factors. The results also provide useful information for the construction of mechanical models of the behavior. Successful models should include functional components corresponding to the identified adaptive processes. In designing the mechanism, we must pay attention not only to the components themselves but also to their interactions, as they are critical for producing the observed behavior.

## 4. Constraining the space of mechanistic models

Goffin’s cockatoos are complex animals that exhibit intricate behavior. This is especially the case in environments that offer many affordances, as is the case for the lockbox. Given this complexity, it is not promising to try to derive a single, highly detailed mechanistic explanation from limited data. However, a more attainable goal is to instead extract constraints on possible mechanisms. Our proposed strategy is to build up an increasing set of such constraints across multiple studies. It is important to note that at our current state of knowledge, we cannot explicitly formulate the set of candidate models yet. This set of candidate models will have to co-evolve with the set of constraints, but as we gather more constraints, it will also become increasingly feasible to formulate a set of candidate models.

In the following, we will relate the descriptive results from the previous section to properties of mechanisms that could account for them. We will explore how the observations we’ve made can render certain mechanistic explanations more plausible than others.

### 4.1. Constraint A—adaptation in multiple interacting factors

Our decompositional analysis illustrates that not only did *T* decrease for each bird from the first to the last solution, but each of the three factors *T* = *E*∗*M*∗Δ also individually decreased (non-monotonously) for each bird. An algorithmic model that explains the evolution of *T* should also provide an explanation for the adaptation observed in each of those factors. Additionally, a robust model should also explain how these factors interact. While each factor contributed linearly to *T* for a single task solution, it is likely that these factors interact and evolve non-linearly over the course of the experiment. This does not necessarily mean that a mechanistic model must implement such a factorization, but an analytical treatment of a mechanistic model must yield similar results as the one we have observed in the birds.

### 4.2. Constraint B—slow and fast adaptation

The birds exhibited both rapid and gradual adaptation in various aspects of their behavior. Specifically, time-to-solution *T* showed a significant and swift improvement in the initial few solutions for all birds. This steep decline in *T* can be explained by similarly steep decline in several of the proposed factors. But additionally we can also observe slow, long-term adaptation in the birds’ capability to target functional parts of the lockbox (decreasing *M*). A suitable algorithmic model should exhibit both trends. It should allow for fast adaptation in each of the factors we proposed, even based on just a single successful trial. However, it should also enable the agent’s behavior to steadily adapt over the course of many sessions. In our interpretation, such fast adaptation based on only a few trials indicates that incremental reinforcement learning models are an unlikely explanation. These typically require many trials to significantly improve an agent’s performance and fast learning may be better explained by learning mechanisms based on episodic memory (Botvinick et al., 2019).

### 4.3. Constraint C—regress to old solution behavior and non-monotony

Two out of the three birds (Muki and Zozo) displayed a temporary decline in wheel-directed actions after their initial success. This represents the most notable example of a non-monotonic adaptive pattern in our data. Additionally we observed that other variables also did not follow a strictly monotonous increase or decrease. Algorithmic models should replicate such re-emergence of behaviors that were temporarily suppressed. In our interpretation, re-emergence could potentially be explained by a reinforcement learning agent’s erroneous credit assignment. As the birds indeed had to touch the door and bar prior to receiving the cashew reward, a discounted reinforcement scheme could be the driving mechanism behind this re-emergence.

### 4.4. Constraint D—Old strategy on new lockbox

At the outset of the experiment, the birds primarily acted on the new lockbox (with wheel) as if it was the old lockbox from the habituation training (without the wheel). Only when this behavior did not lead to movement or success, they started to exhibit more varied behavior, including exploration of the wheel. A possible interpretation is that the birds applied the strategy they learned during the habituation phase because they may not perceive a significant alteration in the lockbox sequence or kinematics. This would imply that mechanisms involving a high degree of physical or kinematic reasoning are unlikely explanations, as they could allow the agent to immediately recognize that the wheel obstructs the motion of the bar. It also indicates that mechanisms with sensitive novelty detection on the appearance of the lockbox are improbable explanations.

### 4.5. Constraint E—Inter-individual differences

The individuals in our study adapted in different ways to solve the task. For instance, the steep decrease in time-to-solution *T* for Muki can be primarily attributed to an improvement in mechanical skill, whereas for Fini, the most suitable explanation for this decrease involves a combination of changes in targeting of actions and engagement. Mechanical models must either incorporate randomness in their adaptation process or initialization to allow for such distinctive adaptive trajectories.

### 4.6. Using constraints

It is unlikely that a single experiment, or even a limited number of experiments, can provide sufficient information to infer the complex, interacting processes giving rise to the observed problem solving behavior. As there is not enough data available for such inference, currently and in the foreseeable future, we will have to establish incremental methods to make coarse inference about mechanisms. The above constraints should serve as a starting point of our interdisciplinary approach to iteratively identify the mechanisms underlying Goffin’s cockatoos mechanical problem solving. We aim to grow this set of constraints to continuously narrow down the set of plausible mechanisms, such that we can then suggest concrete mechanisms with sufficient certainty.

Such an approach requires us to understand how properties of mechanisms map to high-level, abstract descriptions of behavior. To better understand this mapping, it is helpful to apply biological research methods to artificial systems, like robots (similar to Lazebnik, 2002; Jonas & Kording, 2017) because robotic systems offer full introspection into the underlying ground truth mechanisms meaning we can link high-level descriptions of behavior with their generating mechanisms. When we better understand this link, it might also help us infer biological mechanisms from data.

## 5. Conclusion

Our motivation was to close the gap between observations of behavior and implementable, mechanical models. This gap arises because the observational data available does not suffice to identify a unique and matching mechanistic model. We described two methodological approaches to address this issue: a decompositional analysis of behavior and the successive identification of constraints on mechanisms.

A suitable decompositional analysis can inform mechanistic models by providing functional components that may be part of such models and by opening up the possibility to analyze the interaction mechanisms between these components. In our analysis of problem solving behavior in cockatoos, we identified three components: Engagement, mechanical skill, and targeting of contact actions. Each of these had an impact on time-to-solution, but in a different way for different animals and also for different solutions of the same animal. Muki, Fini, and Zozo each progressed differently in the measures *M*, *E*, and Δ. These findings emphasize that a decompositional analysis is indeed crucial. In complex tasks, such as the lockbox, adaptation and task-solving requires heterogeneous processes to interact and analysis should be performed in a way that allows to understand such interaction. Although we are not the first to apply such a decompositional analysis (Biondi et al., 2022), we believe it is important to advocate this approach, as it will help identify building blocks for mechanical models.

Because the gap between available behavioral data and implementable mechanical models is still too large, we did not infer a concrete mechanical model but instead provided constraints on models. Such constraints narrow down the set of possible mechanistic models and represent it *implicitly*. This intermediate step is crucial to advance towards mechanical models without prematurely defining a model based on limited data. For example, we cannot yet infer the concrete mechanisms underlying the animals behavioral changes, but we could gather information that constrains the search for such models. Amongst other constraints, we found (a) fast adaptation, which indicates learning mechanisms using episodic memory might be driving adaptation, but at the same time we found (b) slow learning which may indicate that slower, statistical learning may also play a role, and (c) we observed re-emergence of previously extinguished behavior. These phenomena may be explainable by learning mechanisms based on reinforcement, but we need to gather more data to precisely identify the concrete mechanisms. The constraints we present are not as detailed as a full algorithmic model would be. However, they are a starting point to bridge between the available data and mechanical models. This set of constraints is not final and is subject to change. We plan to co-evolve it with concrete mechanical models of mechanical problem solving in Goffin’s cockatoos in the future.

As an interdisciplinary team of behavioral biologists and roboticists, we realized that the gap between experimental data and concrete model algorithms is currently too large. We believe that identifying constraints on mechanisms is a plausible and important step to fill this gap. On this road, we will not just have to gather additional constraints but will also have to further develop the methodology we employ.

## Note

1.
https://osf.io/jqutx/?view_only=66f513d6748546a195d9978f4178
